# OPA1 deficiency accelerates hippocampal synaptic remodelling and age-related deficits in learning and memory

**DOI:** 10.1093/braincomms/fcaa101

**Published:** 2020-07-15

**Authors:** Ryan J Bevan, Pete A Williams, Caroline T Waters, Rebecca Thirgood, Amanda Mui, Sharon Seto, Mark Good, James E Morgan, Marcela Votruba, Irina Erchova

**Affiliations:** 1 School of Optometry and Vision Sciences, Cardiff University, Maindy Rd, Cardiff, CF24 4HQ, UK; 2 Department of Clinical Neuroscience, Division of Eye and Vision, St. Erik Eye Hospital, Karolinska Institutet, Polhemsgatan 50, 112 82 Stockholm, Sweden; 3 School of Psychology, Cardiff University, Tower Building, 70 Park Place, Cardiff, CF10 3AT, UK

**Keywords:** OPA1, optic atrophy, memory, hippocampus, ageing

## Abstract

A healthy mitochondrial network is essential for the maintenance of neuronal synaptic integrity. Mitochondrial and metabolic dysfunction contributes to the pathogenesis of many neurodegenerative diseases including dementia. *OPA1* is the master regulator of mitochondrial fusion and fission and is likely to play an important role during neurodegenerative events. To explore this, we quantified hippocampal dendritic and synaptic integrity and the learning and memory performance of aged *Opa1* haploinsufficient mice carrying the *Opa1^Q285X^* mutation (B6; C3-*Opa1^Q285STOP^*; *Opa1^+/−^*). We demonstrate that heterozygous loss of *Opa1* results in premature age-related loss of spines in hippocampal pyramidal CA1 neurons and a reduction in synaptic density in the hippocampus. This loss is associated with subtle memory deficits in both spatial novelty and object recognition. We hypothesize that metabolic failure to maintain normal neuronal activity at the level of a single spine leads to premature age-related memory deficits. These results highlight the importance of mitochondrial homeostasis for maintenance of neuronal function during ageing.

## Teaser

Mitochondria generate energy required for memory function. Aged mice having genetically reduced amount of OPA1 protein necessary for energy production have subtle but persistent deficits in behavioural memory. Anatomical examination of the relevant brain region (hippocampal CA1) revealed changes affecting communication between cells and leading to premature age-related memory deficits.

## Graphical Abstract

**Graphical Abstract:**
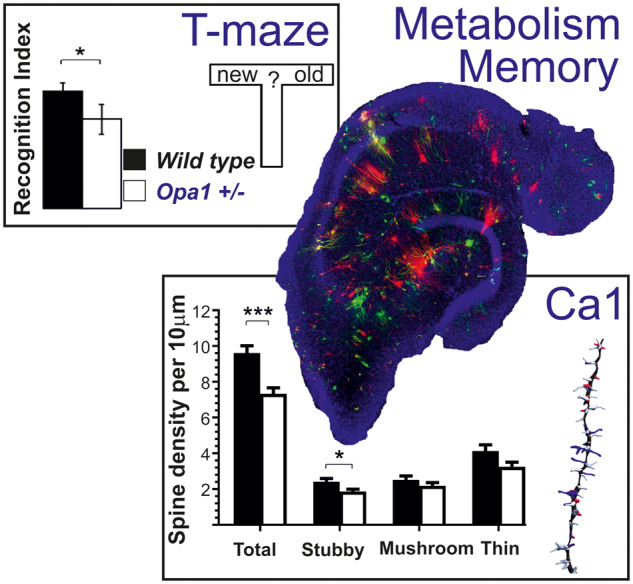

## Introduction

Mitochondria play a critical role in cell death and survival (Fieni *et al.*, 2012). Compromised mitochondrial function (Hedskog *et al.*, 2013) is common in neurodegenerative diseases such as Alzheimer’s, Parkinson's, and Huntington's disease (Schon and Area-Gomez, 2013; Krols *et al.*, 2016; Cai and Tammineni, 2017; Erpapazoglou *et al.*, 2017). While neuronal metabolism accounts for ∼20% of the body’s total energy demand (Yu *et al.*, 2018), mitochondrial respiration, which is least 15 times more efficient at ATP production than glycolysis (Rich, 2003), is largely responsible for meeting this demand. Mitochondrial morphology is controlled by mitochondrial fission and fusion, and in neurons this ensures the correct shape and distribution of mitochondria in relation to dendrite and synapse location. Elongated mitochondria, for example, accelerate the propagation of local changes in membrane potential within a neuron (Skulachev, 2001). Neurons require large amounts of ATP to maintain ionic gradients across cell membranes and support synaptic transmission. The level of ATP demand is specific for each individual spine and synapse. Not surprisingly, many age-related neurodegenerative diseases are characterized by deficient mitochondrial renewal or dynamics as reviewed by Chan (2006). Healthy ageing requires enough metabolic capacity to cope with stress and injury (Atamna *et al.*, 2018). By contrast, reduction in flexibility of cellular transport or inability to meet peak-time metabolic demands results in cellular oxidative stress, senescence, cell type transformation, and, ultimately, cell death (Chauhan *et al.*, 2014; Rattan, 2015).

The mitochondrial fusion protein OPA1 is embedded in the mitochondrial intermembrane space and controls the inner membrane structure (Griparic *et al.*, 2004). *OPA1* mutations result in abnormal mitochondrial dynamics (Liao *et al.*, 2017) and neurodegeneration (Alexander *et al.*, 2000; Delettre *et al.*, 2000). The associated disease, autosomal dominant optic atrophy, is characterized by progressive bilateral central vision loss and is accompanied by extra-ophthalmological symptoms in ∼20% of cases. The autosomal dominant optic atrophy disease phenotype can be replicated in animal models (Williams *et al.*, 2011; Carelli *et al.*, 2013; Ham *et al.*, 2019). Heterozygous B6:C3-*Opa1*^Q285STOP^ (*Opa1^+/−^*) mice have normal longevity, but gradually develop visual dysfunction, resembling a slow onset model of the human disease (Davies *et al.*, 2007). The *Opa1^+/−^* mutation results in a ∼50% reduction in OPA1 levels, but does not alter its structure (Davies *et al.*, 2007). The initial retinal pathology in *Opa1^+/−^* mice; retinal ganglion cell dendritic degeneration, synaptic loss and mitochondrial changes, starts at 10 months, but the visual phenotype is only detectable clinically by 12 months (Davies *et al.*, 2007; Williams *et al.*, 2010). These deficits can be quantified by visual electrophysiology (electroretinography; Barnard *et al.*, 2011) and visual acuity [optokinetic response (OKN) behavioural testing; Davies *et al.*, 2007]. *Opa1* haploinsufficiency reduces mitochondrial efficiency (Del Dotto *et al.*, 2018), but the associated pathology is relatively mild in comparison with mutations causing protein misfolding or truncation. In humans, OPA1 protein levels decrease with age causing cases of muscle dystrophy (Tezze *et al.*, 2017) or even systemic neurodegeneration (Sarzi *et al.*, 2012).

In this study, we assessed hippocampal-dependent memory (Tulving *et al.*, 1972) using two tests that could be performed with reduced visual acuity and complement each other (Denninger *et al.*, 2018): one evaluates spatial learning and the other non-spatial learning of object identity. While all our mice were able to perform behavioural tasks, *Opa1^+/−^* mice showed some consistent deficits. Following this finding, we examine cellular integrity of CA1, as both tests require intact CA1 hippocampal region.

## Materials and methods

### Animals and genotyping

All experiments were conducted in accordance with the UK Animals (Scientific Procedures) Act and the Association for Research in Vision and Ophthalmology (ARVO) guidelines for the use of animals in research. The Cardiff University Biological Standards Committee approved the experimental protocols. We followed the guidelines of the Animal and Plant Health Agency for compliance with Regulation (EC) 1069/2009 and implementing Regulation (EC) 142/2011 for the transport, storage, use and disposal of animal by-products.

The mouse model (B6; C3-*Opa1^Q285STOP^*; *Opa1^+/−^*) has been described in detail elsewhere (Davies *et al.*, 2007; Williams *et al.*, 2011). The colony was kept in a 12-h light (10 lux)/12-h dark cycle with food and water available *ad libitum*. All experimental animals were outcrossed to a C57BL6J background to at least five generations. Visual acuity was evaluated by behavioural testing (OKNs) at 14 months of age followed by two memory tests [spatial novelty testing (T-maze) and novel object recognition (NOR) test] at 15 months. An additional control group of animals was tested for visual acuity at 20 months.

All mice taking part in the behavioural memory tests were euthanized at 16 months of age. The genotype was confirmed, and neuronal structure in hippocampal tissue examined by DiOlistics (WT; *n* = 5, *Opa1^+/−^*; *n* = 6), RT-PCR (WT; *n* = 3, *Opa1^+/−^*; *n* = 3), western blotting (WT; *n* = 2, *Opa1^+/−^*; *n* = 2) and immunohistochemistry (WT; *n* = 5, *Opa1^+/−^*; *n* = 6) to obtain pilot data. We followed up the initial study with a more robust examination of hippocampal tissue (reported here) from 24 mice (16 months of age) with DiOlistics (WT: *n *= 6; *Opa1^+/−^*; *n* = 6, male/female ratio was 50/50) and western blots (WT; *n* = 5, *Opa1^+/−^*; *n* = 7).

### Behaviour

All behavioural tests were performed in a dedicated behavioural lab. Animals were housed in home cages before and after the procedure; all animals were returned to the home cages for at least 3 min between trials and for at least 1 min between different phases of the same test.

#### Visual acuity tests

We followed the EMPReSS method (http://empress.har.mrc.ac.uk/) to record OKNs. We tested visual acuity using drifting, full contrast black-and-white square wave gratings (2°, ∼1 cm width) with spatial frequency of 0.25 cycles per degree selected as the known upper limit of spatial frequency for aged *Opa1^+/−^* mice (Davies *et al.*, 2007). All mice (WT: *n* = 12, *Opa1^+/−^*: *n* = 12, 6 animals per group at 14 and 20 months) were habituated to the apparatus (optokinetic drum, with stimuli presented on the inner surface) for 10 min in the dark, then 5 min in light. The illuminance was 250 candelas at the level of the platform. The drum rotated clockwise for 1 min, and after a 30 s rest period, reversed the direction. Each mouse ran the test daily for 3 days in a row, three times a day. We filmed and scored mice behaviour using two independent experts masked in relation to mice age and genotype. We counted the number and duration of episodes when head movements followed the direction and speed of rotating drum (Davies *et al.*, 2007).

#### Memory tests

We selected memory tests previously suggested for subjects with impaired vision (Brown and Wong, 2007; O’Leary *et al*., 2011; Wolf *et al*., 2016): T-maze and a simple variant of the NOR. We purposefully avoided tests based on visually guided navigation such as large open arena or Morris water-maze. While both selected tests exploit the inherent preference of mice for the novelty, they are complimentary regarding brain circuitry involved in the task (Denninger *et al.*, 2018). The T-maze evaluates spatial learning, which relies heavily on hippocampus. The NOR evaluates non-spatial learning of object identity, which relies on multiple brain regions. Each mouse ran the test multiple times on different non-consecutive days.

##### Spatial novelty (T-maze) test

We followed standard protocols for testing spatial novelty in mice using a T-maze. All mice (WT: *n* = 10, *Opa1^+/−^*: *n* = 16) were kept in their holding cages before, after and during the testing. Initially, one arm of a T-maze was blocked off and mice were allowed to investigate the maze for 10 min. The door to the blocked off arm was then removed and mice were allowed further 5 min for active exploration. There was only a minimum delay between ‘sampling’ and ‘choice’ phases of the test. Time spent in the base of the maze (TB, also referred as ‘start’), and in each of the arms [novel (TN), also referred as ‘new’ and familiar (TF), also referred as ‘old’] was recorded. Mice were tested for six consecutive days, at the same time of day on each occasion. Standard discrimination (DI) and recognition indices (RI) measured novelty behaviour.

(eq.1) DI=(TN-TF)/(TN+TF)

 

(eq.2) RI=TN/(TN+TF)

A positive discrimination index score indicates a preference for novel. The RI score indicates the proportion of time spent investigating novelty relative to the total exploration time.

##### Novel object recognition test

We followed a previously published protocol (Ennaceur and Delacour, 1988) with minor modifications. All mice (WT: *n* = 9, *Opa1^+/−^*: *n* = 18) were weighed before testing and kept in their holding cages before, after and during the testing. Mice were first habituated to an empty white walled Perspex arena (60 cm × 60 cm × 15 cm) for 10 min on each of three consecutive days. Each mouse was then placed in the arena with two identical objects for 10 min and allowed to explore (exposure phase). Time spent exploring different objects at exposure phase was noted and is further referred to as ‘contact time’. After 1 min in the home cage, the mouse was placed back in the arena which now contained one original and one novel object. A mouse was allowed further 5 min of exploration (test phase). The time spent exploring the arena base (TB), familiar (TF) and the novel (TN) objects during test phase was recorded. Mice ran the test for six consecutive days, at the same time of day on each occasion. Objects (*n* = 7) made from different materials (metal, resin, silicone, wood, fabric, slate and glass) were alternated on each day to prevent any possible bias. As with the T-maze, we calculated discrimination index (eq.1) and RI, (eq.2).

#### Statistical analyses of behavioural data

Time variables in all behavioural tests (average time for following rotation drum in OKN visual acuity test, TB, TN and TF in both T-maze and NOR) were normally distributed and were analysed by parametric statistics. Count values (number of head turns) in OKN, and number of correct choices in T-maze and NOR were not necessary normally distributed, especially when counts were small. Recognition indices (range values) were not normally distributed (skewed unimodal distributions). All not normally distributed data were compared using non-parametric statistics: Kruskal–Willis test as alternative to the one-way ANOVA and the Mann–Whitney is an alternative to the two-sample *t*-test. To increase the power of the statistical tests, each subject/sample was tested several times and data pooled for the test (with additional adjustment for effective sample size). The between-subject factors and the effect sizes are indicated together with *P*-values. All plots are presented as mean and standard error. Significance levels marked with *, **, *** for *P*-values 0.05, 0.01 and 0.001 accordingly.

### Tissue analysis

For all tissue analysis presented in this study, the experimenters were masked in relation to mice genotype (published methods Davies *et al.*, 2007) to avoid any possible bias in data processing and analysis.

#### Quantitative RT-PCR

Tissue analyses were performed by Central Bioscience Services, Cardiff University. Total RNA was isolated with Trizol-Reagent and purified using the RNeasy Clean Up kit (Qiagen). One microgram of total RNA from each sample was reverse transcribed using the high capacity cDNA reverse transcription kit (Applied Biosystems). Quantitative PCR reactions were prepared using TaqMan^®^ Universal PCR Master Mix, No AmpErase^®^ UNG (Applied Biosystems) and mixed with cDNA, Taqman primers and probe gene specific assay mix. TaqMan gene expression assays (Applied Biosystems) were used for *Mus musculus* Opa1 (Assay ID: Mm00453879_m1), Syp (Mm00436850_m1) and PSD95 (Mm00492193_m1). qPCR was performed using an ABI Prism 7900HT (Applied Biosystems). Assays were carried out in triplicate with the mean Ct values used to calculate the relative gene expression levels after normalizing to 18S RNA levels (endogenous control: VIC/MGB Probe, Primer Limited). Analysis of relative gene expression data was performed using the 2^−ΔΔ^^*CT*^ method. Statistical analysis was performed on normally distributed ΔΔC values by multivariate ANOVA with Tukey *post hoc* test. The data are represented as RQ values in folds.

#### Western blot analysis

We tested two synaptic proteins (PSD95 and synaptophysin), microtubulin protein Tau, and OPA1 protein simultaneously on the same samples to correlate protein changes. The nature of synaptic changes can be interpreted as following: (i) reduction in synaptophysin with no changes in other synaptic proteins would indicate reduced efficacy of pre-synaptic terminal; (ii) reduction in synaptophysin with significant reduction in Tau would signal axonal degeneration; and (iii) PSD95 reduction in combination with modest decline in Tau would signal reduced size or postsynaptic terminals. Since OPA1, PSD95 and Tau had similar molecular weights (∼80kDa) and bands in close proximity, membranes had to be stripped and washed several times to optimize band separation for quantitative analysis (a range of exposure times were used to optimize the appearance of each protein band).

For total protein extraction, mouse hippocampus was sonicated in RIPA buffer containing 1% ethylenediaminetetraacetic acid free protease inhibitors (Roche) on ice. Homogenized hippocampal samples with loading buffer were pre-heated to 95°C for 5 min. Equal amounts (40 μg) of protein extracts were separated by 12% sodium dodecyl sulphate-polyacrylamide gel electrophoresis (SDS-PAGE) in Tris/glycine/sodium dodecyl sulphate running buffer (Biorad, Hemel Hempstead, UK). Electrophoresis was run for 1 h 15 min at 100 V; then gels were transferred to 0.2 μm nitrocellulose membrane (Bio-Rad, UK) for 75 min at 250 mA in a cold Tris/glycine transfer buffer (Biorad, Hemel Hempstead, UK) with 20% methanol (v/v). After the transfer, membranes were blocked with 5% bovine serum albumin (BSA, Sigma) in phosphate buffered saline for 1 h at room temperature. Precision Plus Protein Standard (Bio-Rad, UK) was loaded in a volume of 10 μl/lane to show the location of proteins at the distinct size in the gel.

To quantify and distinguish proteins with similar molecular weights we used Gel Stripping by Low pH (2.2) buffer (glycine, sodium dodecyl sulphate and Tween20 in distilled water). After reading out the results of the first set of proteins, gels we incubated in buffer two times, washed in TBS and TBST, and blocked in 5% skimmed milk (1 h). The gels were then re-probed with new primary antibody overnight. Proteins were probed with mouse anti-Tau (ab80579, 1 µg/ml, 1:1000), rabbit anti-synaptophysin (ab14692 1:1000), rabbit anti-PSD95 (ab18258, 1 µg/ml, 1:1000) and rabbit monoclonal to OPA1 (ab157457, 1:1000) (all Abcam, Cambridge, UK) overnight at 4°C. The mouse monoclonal anti-β-actin antibody (A5441, clone AC-74, 1:2000) (Sigma) and mouse monoclonal VDAC1/Porin antibody (ab14734, 1:2000) (Abcam, Cambridge, UK) were used as a loading control.

Following four washes in phosphate buffered saline, rabbit anti-mouse and goat anti-rabbit horseradish peroxidase secondary antibodies (Sigma) were used at 1:1000 and 1:2000, respectively, at room temperature for 2 h before being membrane being developed using ChemiDoc XRS+ imaging system with Image Lab image.

The membranes were cut into two parts; one for the high molecular weight proteins (OPA1, PSD95 and Tau) and one for the smaller molecular weights (β-actin and synaptophysin) to allow optimal transfer conditions.

Bands from multiple western blots were quantified using Bio-Rad Image lab software. We evaluated consistency of both normalization proteins (voltage-dependent anion channel and β-actin) as a function of OPA1 levels. All values were therefore normalized to β-actin because its single band was easily detected and well separated from other bands.

The protein data were normally distributed and compared with parametric statistics. The between-subject factors and the effect sizes (-partial- eta squared values) are indicated together with *P*-values. All plots are presented as mean and standard error, the significance levels marked with *, **, *** for *P*-values 0.05, 0.01 and 0.001 accordingly.

#### DiOlistic cell labelling

Cells were labelled randomly with the density of labelled cells depending primarily on the dye concentration in the bullets. We used a previously published protocol (Williams *et al*., 2013; Binley *et al*., 2016; Williams *et al*., 2016) with a few alterations. In brief, hippocampi were isolated under a dissection microscope and transferred to a McIlwain Tissue Chopper for 200 µm slicing in a septal to temporal direction. To increase the yield of labelled neurons without affecting our ability to trace individual cells, we used two different dyes. Tungsten particles (1.7 µm, 100 mg: BioRad) were coated with 2 mg of DiI (Molecular Probes) and 4 mg of DiO (Molecular Probes) distributed in Tefzel tubing and cut into 1.2 cm ‘bullets’. Hippocampal tissue slices were labelled (two labelling colours green and red) with a Helios gene gun at 120 psi through a 3 µm polyethylene terephthalate membrane filter to prevent dye particle clumping. The total time from dissection to labelling was less than 10 min. Slices were then incubated in Neurobasal Medium (Sigma) at 37°C, 5% CO_2_ for 30 min to allow dye diffusion. Following incubation, the tissue was transferred to adhesive microscope slides (HistoBond, Fisher Scientific), fixed in 4% paraformaldehyde for 30 min and counterstained with Hoechst nuclei. Slides were mounted in Prolong Gold AntiFade Reagent (Invitrogen) and imaged with confocal microscope within 1 week.

##### Confocal imaging

Though cells were labelled in all hippocampal regions, our analysis was restricted to CA1 pyramidal neurons. Neurons were imaged at 20× at 0.7 magnification of visual field for analyses of dendrite structure (Z stack interval 1 µm). Selected dendrites were imaged at 40× at full magnification to analyse spine structure (Z stack interval 0.6 µm). We imaged on average at least nine well labelled neurons per subject.

##### DiOlistic data analysis

Cell dendrites and spines were traced using Imaris 9.2 software (Bitplane, Oxford Instruments). Unprocessed confocal Z stack images were reconstructed in 3D to generate Sholl plots of dendritic complexity (Sholl, 1953) and automated analyses of dendritic branching patterns. All dendritic spines were automatically counted using the FilamentTracer Spine Classifier extension in Imaris, based on their shape (stubby, mushroom and thin). Spine counts were normalized to the length of the imaged dendrite. Proximal and distant apical dendrites were analysed separately. We used combination of parametric and non-parametric statistics. Similar to behavioural data, all cells from all subjects were pooled for the statistical test (with additional adjustment for effective sample size).The between-subject factors and the effect sizes are indicated together with *P*-values. All plots are presented as mean and standard error, the significance levels marked with *, **, *** for *P*-values 0.05, 0.01 and 0.001 accordingly.

### Data availability

Original and derived data supporting the findings of this study are available from the authors on request.

## Results

### Vision loss in *Opa1* deficient mice (B6; C3-*Opa1*^Q285STOP^, *Opa1*^+/−^)

We assessed severity of visual deficits using OKNs prior to memory tests (at 14 months) and at 20 months. When an animal resolves the stimulus pattern on the inner wall of optokinetic drum, it follows the movement of the drum with its head to maintain fixation; this reflex response does not depend on previous exposure or training (Thaung *et al.*, 2002). The scores for this visual acuity test were not directly comparable between different ages. Young animals followed the stimulus often, but for a very short time. In contrast, the older animals followed the stimulus for longer with fewer head turns. To avoid any bias, we used both measures and only directly compared animal performance within the same age group.

**Figure 1:**
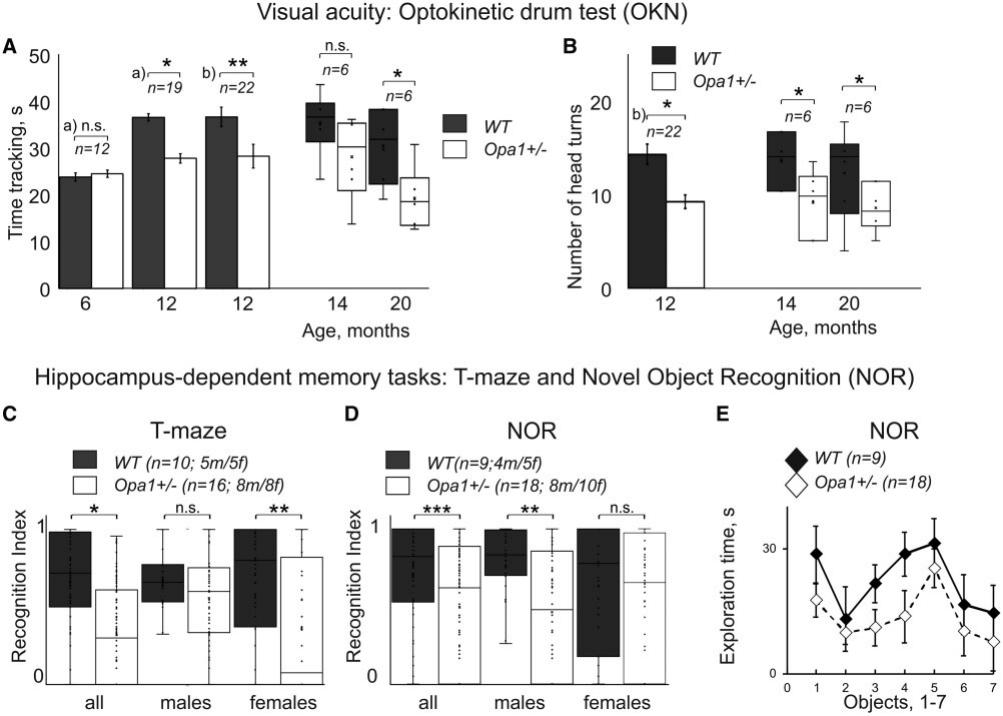
**Summary of mice behavioural data.** (A, B) Two measures of mice visual acuity using reflex responses in optokinetic drum (OKN) at different ages. The *n* represents the minimum number of animals per group for a given experiment. (**A**) Total time tracking drum rotation as a function of genotype and animal age. Data obtained in this study are depicted as whisker plots and previously published data for younger age (a) and Davies *et al.*, 2007; b) Smith *et al.*, 2016) are shown as bar plots. (**C, D**) Recognition index in hippocampus-dependent learning and memory tasks at 15 months of age. (**C**) T-maze. (**D, E**) Novel object recognition (NOR) task. Data are shown for two genotypes for all animals and separately for males and females. The *n* represents the number of animals per group for a given experiment; the male/female ratio was 50/50, the exact numbers are specified for each experiment. (**E**) Difference in exploration time between seven different objects used in NOR task (averaged for all animals). All data are presented as mean and SE, significance levels marked with *, **, *** for *P*-values 0.05, 0.01 and 0.001 accordingly. *T*-test was used to compare groups for OKN time tracking, and TB, TN and TF values in T-maze and NOR; Mann–Whitney U-test was used to compare values that were not normally distributed (number of head turns in OKN task, RI in T-maze and NOR). Meta-analyses of visual acuity data is shown in Supplementary Fig.1. Additional data for the learning and memory tests are shown in Supplementary Fig. 2.

*Opa1^+/−^* mice demonstrated a reduction in the number of tracking episodes and in tracking time per episode compared to age-matched controls (Fig. 1A and B, left; head turns reduced from 13.3 ± 2.1 to 9.8 ± 0.9 at 14 months, *Z* = −1.84, *P *=* *0.033; and from 10.8 ± 1.9 to 7.4 ± 1.1, *Z* = −1.68, *P *=* *0.046 at 20 months; total tracking time reduced from 32.4 ± 2.7 to 25.6 ± 2.5 at 14 months, *Z* = −1.63, *P *=* *0.052 and from 27.7 ± 2.8 to 17.3 ± 1.9, *Z* = −1.88, *P *=* *0.03 at 20 months of age).

Although the visual acuity in aged *Opa1^+/^*^−^ mice was reduced compare to aged matched controls, animals could still see relatively high special frequencies at high contract (0.25 cycles per degree; corresponding to 1 cm wide contrast line at about 20 cm viewing distance). Given that lower spatial frequencies (from 0.02 to 0.04 cycles per degree) excite the most of the primary visual cortex neurons in mice (Ayzenshtat *et al.*, 2016); and a mean optimal spatial frequency in V1 is ca. 0.15 cycles per degree (Niell and Stryker, 2008), we concluded that mice preserved enough vision to manage behavioural performance in memory tasks.

In addition, we performed meta-analyses of visual acuity tests combining results of experiments performed in the current study with previously published results (Davies *et al.*, 2007) where visual acuity tests were performed in the younger mice in the same behavioural laboratory and with the same methodology. The results are detailed in the Supplementary Fig. l. The between-subjects’ factors (gender, age and genotype) were analysed with multivariant ANOVA on combined sample of 123 animals. The genotype emerged as the most significant factor (*P* < 0.001, *F* = 12.4; *η*^2^ = 0.08, df = 1), followed by age/genotype interaction (*P* = 0.004, *F* = 4.6; *η*^2^ = 0.09). Gender or gender/age, gender/genotype interactions were not detected as significant factors in relation to vision loss when total time of stimulus tracking was considered. However, analyses of individual tracking episodes showed that males and females used different behavioural strategies to compensate for vision failure (*P* < 0.001, *F* = 11.3; η^2^ = 0.06): when first signs of vision deficits appeared, females opted for more short tracking episodes while males increased duration of each tracking episodes. The total time spent tracking stimulus was not different between the genders.

### Learning and memory

The T-maze test based on the willingness of rodents to explore a new environment (reviewed in Dudchenko, 2004). When novel arm was open for the first time, all animals spent some time at the base of the maze (TB) exploring/hesitating before entering a maze arm. The data are provided in full in Supplementary Fig. 2A–C. For wild type (WT) animals, TB just exceeded 10 s [all: 11.9 ± 1.9 s; M (males): 13.6 ± 4.1 s; F (females): 11.0 ± 2.6 s] and animals chose the novel arm in ∼70% of trials (all: 71.6%; M: 66.7%; F: 74.4%). Some animals that initially had chosen the familiar arm later returned to investigate the novel arm (all: 28.4%; M: 100%; F: 36.4%). This returning behaviour differed between sexes, with males usually visiting both arms on each trial. The RI (Fig. 1C) represents a proportion of time spent in a novel part of the maze and did not differ between the groups (all: 0.71 ± 0.2; M: 0.69 ± 0.3; F: 0.72 ± 0.3). All mutant animals spent more time (TB) exploring/hesitating before making a choice (all: 23.1 ± 2.3 s; M: 17.2 ± 2.5 s; F: 31.7 ± 4.1 s; *F* = 8.62, *P* < 0.01; *η*^2^ = 0.14; ANOVA and Turkey HSD). The difference was significant for both sexes (males: *Q* = 5.2, *P* < 0.01, females: *Q* = 7.4, *P* < 0.01, with additional differences between sexes statistically confirmed *Q* = 4.9, *P* < 0.01). The preference for the novel arm was at the chance level (all: 46.9%; M: 51.8%; F: 40%); the females seemed to prefer familiar rather than novel environment. As in WTs, some animals that had initially chosen the familiar arm, returned later to investigate the novel arm; this behaviour was more prevalent in males (all: 28.1%; M: 81.5%; F: 20.8%). This behaviour helped mutant males to overcome deficits as judged by RI measures (all: 0.54 ± 0.2; M: 0.59 ± 0.2; F: 0.35 ± 0.3; KW and Dunn; H = 14.1; *P* = 0.003; η^2^ = 0.10); with difference confirmed between sexes (T = 2.7; *P* = 0.03; M: T = 1.16; *P* = 0.74; F: T = 2.9; *P* < 0.01).

The RI is shown in Fig. 1C (right); the control data are in good correspondence with the results from an ageing study by Von Bohlen Und Halbach *et al*. (2006).

In novel object recognition test (NOR), all animals spent some time exploring arena before approaching a particular object (TB) and this time was just above 10 s in the WT group (all: 10.7 ± 1.5s; M: 11.0 ± 1.9s; F: 10.4 ± 2.1s). The data are provided in full in Supplementary Fig. 2D–G. WT animals chose novel object in more than 75% of trials (all: 77.7%; M: 79.5%; F: 76.0%). Mutant animals spent more time exploring (TB) before making a choice (all: 30.1 ± 2.1s; M: 29.9 ± 2.8 s; F: 30.4 ± 3.1 s) with preference for the novel object almost at chance level (all: 56.6%; M: 50.9%; F: 66.4%). ANOVA and Turkey HSD (*F* = 12.4; *P* < 0.001; η^2^ = 0.17) attributed this difference to mutation rather than sex difference. All WT animals spent little time investigating familiar objects (TF) (all: 6.7 ± 1.2 s; M: 6.6 ± 1.3 s; F: 6.8 ± 1.8 s) and more time investigating novel objects (TN) (all: 21.5 ± 1.9 s; M: 26.0 ± 2.6 s; F: 18.0 ± 2.6 s). In contrast, mutant males spent similar amount of time investigating both objects [novel (TN): 13.5 ± 1.7 s; familiar (TF): 13.0 ± 1.8 s]; by contract, mutant females spent little time investigating familiar object (TF) (similar to WT), and more time investigating novel objects (TN) (less than WT females) [novel (TN): 12.63 ± 1.7 s; familiar (TF): 6.3 ± 1.7 s]. The differences were confirmed statistically: time for exploration of familiar object increased in males (*F* = 3.7; *P* = 0.011; η^2^ = 0.01; *Q* = 4.09, *P* = 0.02) and time investigating novel objects reduced in all mutants (*F* = 2.7; *P* = 0.047; η^2^ = 0.001) without differences between sexes.

This behavioural pattern was reflected in RI: WT (all: 0.76 ± 0.02 s; M: 0.8 ± 0.03; F: 0.72 ± 0.03) and mutants (all: 0.56 ± 0.01; M: 0.51 ± 0.03; F: 0.66 ± 0.03). Statistical analyses (KW, Dunn) confirms difference between genotypes (H = 14.01; *P* = 0.003; η^2^ = 0.87); with partial effect only confirmed for males (M: T = 3.9; *P* < 0.01; F: T = 0.98; *P* = 0.81). Taken together, our data suggest that both sexes had mutation-related deficit in this task. The observed changes in RI are shown in Fig. 1D (right); our control data are in good correspondence with ageing study by Wahl *et al.* (2018). We estimated contact time for different objects used in this study using measurements of time spent exploring an object during exploration phase (Supplementary Fig. 2F). As Fig.1E suggests, mutant group spent less time exploring objects, but the contact times were not statistically different. For both groups, mean contact time was within the range accepted to be sufficient to form object memory There was no significant difference in time spent investigating individual objects (Fig. 1E), made from different materials (metal, resin, silicone, wood, fabric, slate and glass). Our animal performance in behavioural memory tests indicate subtle but persistent learning and memory deficits in *Opa1+/*− mice that have not been previously reported.

### Anatomy of CA1 region

DiOlistic labelling was used for the rapid structural visualization of large numbers of multiple neurons within blocks of neural tissue.

**Figure 2:**
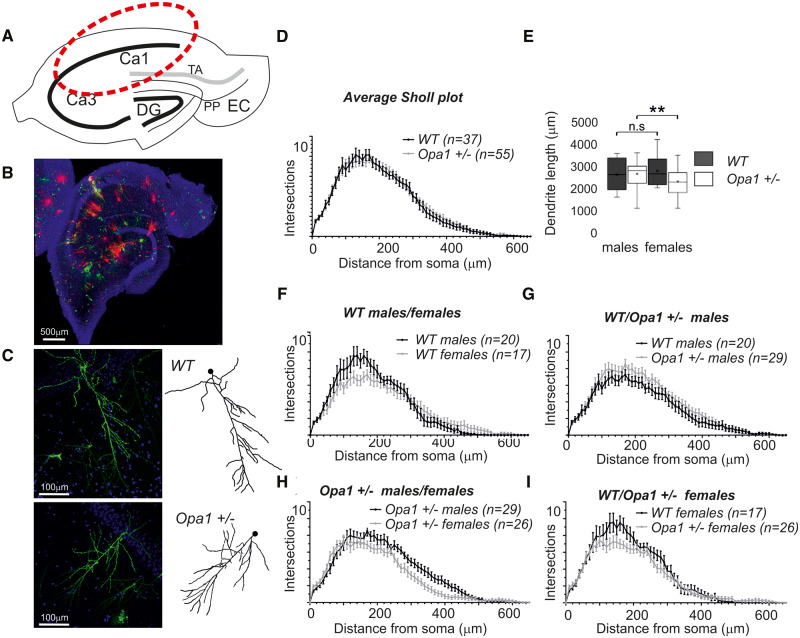
**DiOlistic cell labelling, dendritic tree analysis.** (**A**) Schematic representation of a caudal hippocampal section. Information flow through the hippocampal circuit: sensory inputs from layer II entorhinal cortex (EC) to all fields of the hippocampal formation, including the dentate gyrus (DG) and all CA fields (including CA1 and CA3) is organized via three synaptic perforant path (PP); layer III EC cells project to CA1 via the temporo-ammonic pathway (TA). (**B**) Composite confocal image of DiOlistically labelled hippocampal section. Neurons are labelled red and green by fluorescent dyes. Only CA1 pyramidal neurons were quantified in this study. Cell nuclei are labelled in blue with Hoechst staining. We imaged on average at least nine well-labelled neurons per subject and traced most of the imaged cells. (**C**) Labelled CA1 neuron imaged with confocal microscope ×20 and apical dendrite traced with Imaris software. (WT—top, *Opa1^+/−^* bottom). (**D**) Sholl plots, representing complexity of apical dendrites as a function of distance from the cell body. (**E**) Average dendrite length. The data represented as whisker plots summary of all imaged cells (92 cells in total grouped by gender and genotype, with the smallest group containing 17 cells). The parameter was reduced in female mutants (*P *<* *0.01). (**F, G**) Average Sholl plots for each gender and genotype highlight possible differences in dendrite’s length and complexity. The *n* represents the number of labelled CA1 pyramidal cells analysed for each group. The data were obtained from 12 animals, 6 WT and 6 *Opa1 +/−* (3 males and 3 females in each group). We used non-parametric Kruskal–Willis test to compare results of Sholl plot and average dendrite length. All data are presented as mean and SE, *P*-values <0.05, <0.01, <0.01 are encoded as one, two or three stars accordingly.

Figure 2A shows a schematic representation of a caudal hippocampal section and Fig. 2B a composite confocal image of a DiOlistically labelled section, indicating the quantity and quality of neuronal labelling. Figure 2C gives an example of labelled CA1 neurons and traced apical dendrite from WT and *Opa1^+/−^* mice, respectively. We have analysed on average nine neurons from each subject (range from 2 to 14, three subjects of each gender for each group). Reconstructions of the apical CA1 dendritic field allowed analysis dendritic integrity, Fig. 2D and E. Although some of CA1 neurons in *Opa1 +/−* animals had reduced dendritic complexity, individual variability within each subject was large and on average we did not detect any significant changes in dendritic complexity. We concluded that dendritic integrity is largely maintained in Opa1^*+/−*^ mutants. We also analysed multiple morphological aspects of dendritic structure (data are not shown on the figure) which did not reveal any difference between groups. Such as: individual dendrite lengths (WT 37.23 ± 1.415 µm, *Opa1^+/−^* 38.41 ± 1.040 µm), total dendritic field length (WT 2639 ± 125.6 µm, *Opa1^+/−^* 2610 ± 110.9 µm) or the total number of branch points (WT 36.26 ± 2.030, *Opa1^+/−^* 34.88 ± 1.802). The segmentation of dendritic field based on dendrite branching levels (1^°^ to 5^°^) revealed that the sum of dendritic length at each level was maintained both in close proximity to the cell soma and distal dendrites between mutant and control animals.

In *Opa1^+/−^* female mice the apical dendrites of CA1 neurons were shorter, with the total dendritic length reduced by ∼20% compared to males, from 2896 ± 153.9 µm in males to 2417 ± 133.2 µm in females (Fig. 2E, ANOVA, *P* < 0.01, df = 3, *F* = 4.03, η^2^ = 0.097, with non-equal variance *post hoc* test, *P* = 0.046, between female mutants and controls, *P* = 0.032, between male and female mutants). When analysing branching levels we noted that the sum of primary and secondary branches was also reduced in females compared to males by more than 30% (34% (*p *=* *0.0132) for primary dendrites, and 33% (*P *=* *0.0145) for secondary dendrites). Dendritic lengths at all other levels remained consistent with controls.

**Figure 3:**
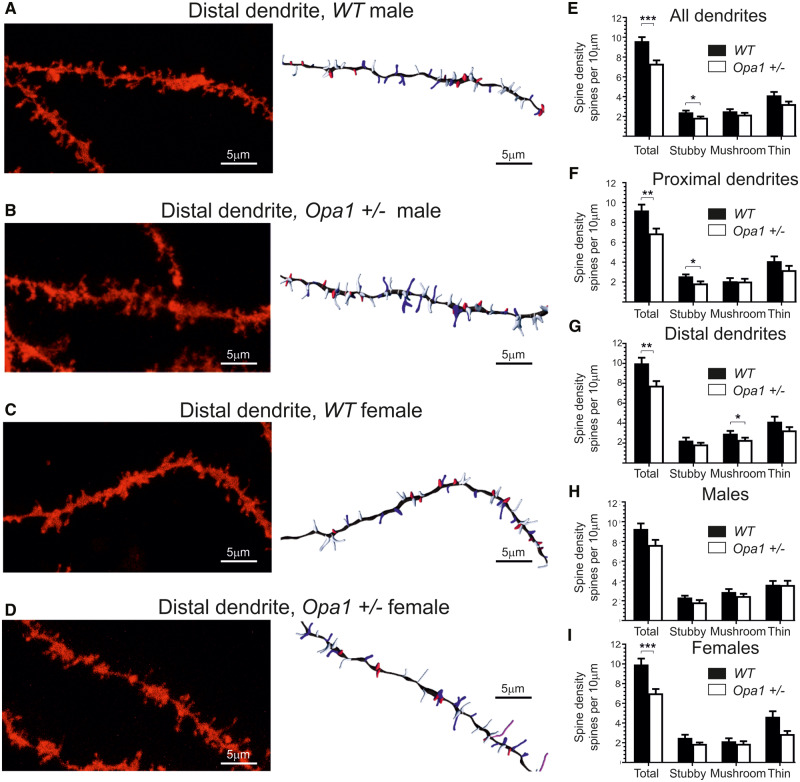
**DiOlistic cell labelling, spine analysis.** (**A–D**) CA1 pyramidal cells apical dendrites imaged with confocal microscope ×40. The right panels represent the same dendrite with spines automatically detected and classified (Imaris). (**A**) A CA1 neuron in WT male. (**B**) A CA1 neuron in WT female. (**C**) A CA1 neuron in *Opa1+/−* male. (**D**) A CA1 neuron in *Opa1+/−* female. (**E**) Average spine density for all spines. (**F,G**) Location differences in spine morphology between distal and proximal regions (**F** Distal, **G** Proximal). (**H,I**) Gender differences in spine morphology for all dendrites (**H**-males, **I**-females). The images (one dendritic segment per labelled neuron) were obtained from a total of 36 different neurons for each genotype (18 dendritic segments corresponded to proximal with the other 18 corresponding to distal, when separated into males and females the total number of dendritic segments analysed totalled 18 for each group). All data are presented as mean and SE. Statistical analysis was performed by non-parametric Kruskal–Willis test; *P-*values *<0.05, **<0.01, ***<0.001.

All spines in the selected regions were classified into filopodia, stubby, mushroom and thin types (Fig. 3A–D, Supplementary Figs 3 and 4). We detected relatively few filopodia and no differences between WT an *Opa1^+/−^* (0.49 ± 0.14 and 0.26 ± 0.08 spines/10 µm, respectively). The relative density was calculated for each type of spine in different parts of the dendritic tree (Fig. 3E–G). *Opa1^+/−^* mutants, both sexes, displayed a 24% reduction (*P *<* *0.001; *T* = 4.27; df = 70; η^2^ = 0.21) in overall spine density on CA1 apical dendrites, Fig. 3E (9.6 spines/10 µm ± 0.41 in WT animals and 7.36 spines/10 µm ± 0.34 in *Opa1^+/−^*). It was noted that both proximal and distant apical dendrites were affected [*P *<* *0.01; *T* = 3.02; df = 34; η^2^ = 0.21 for proximal, *P *=* *0.0089 (two-tailed Mann–Whitney: *Z* = −1.830; η^2^ = 0.09) for distal]]. Proximal dendrites mostly lost stubby synapses (27.8% reduction from 2.579 ± 0.20 in WT to 1.86 ± 0.21 spines/10 µm in *Opa1^+/−^*, *P *=* *0.017; *T* = 2.48; df = 34; η^2^ = 0.15), while distal dendrites mostly lost mushroom spines [21.4% reduction from 2.948 ± 0.27 in WT to 2.316 ± 0.23 in *Opa1^+/−^*, *P *=* *0.049 (two-tailed Mann−Whitney: *Z* = −0.825; η^2^ = 0.02]. We have noticed some gender-related variation in spine loss and conducted multivariant ANOVA analyses to investigate all relevant factor (gender, genotype and spine types). We were able to confirm some of the differences; however, our data were too few to conduct full analyses for all type of spines. The genotype [*P* < 0.001, F(3,272) = 10.4, df = 3, 3% of total variance] and spine type [*P* < 0.001, *F*(3,272)=232.2, df = 3, 67% of total variance] and their interaction [*P* = 0.009, *F*(9,272) = 6.7, df = 3, 2% of total variance] were largely defining the differences observed. *Post hoc* comparison showed that for mushroom and stubby spines, there was not any gender-related differences in spine number (Fig. 3H and I). By contrast, we observed a female only reduction in long thin spines in *Opa1*^+/−^ mice compared to WT (37.8% reductions, *P *=* *0.0086, η^2^ = 0.18), from 4.63 ± 0.56 to 2.88 ± 0.31. Overall, female mutants had almost 30% of spines compare to WT females (*P *<* *0.01; η^2^ = 0.31) reduction from 9.94 ± 0.60 spines/10 µm to 7.01 ± 0.44 spines/10 µm.

#### Synaptic proteins in hippocampal region

**Figure 4:**
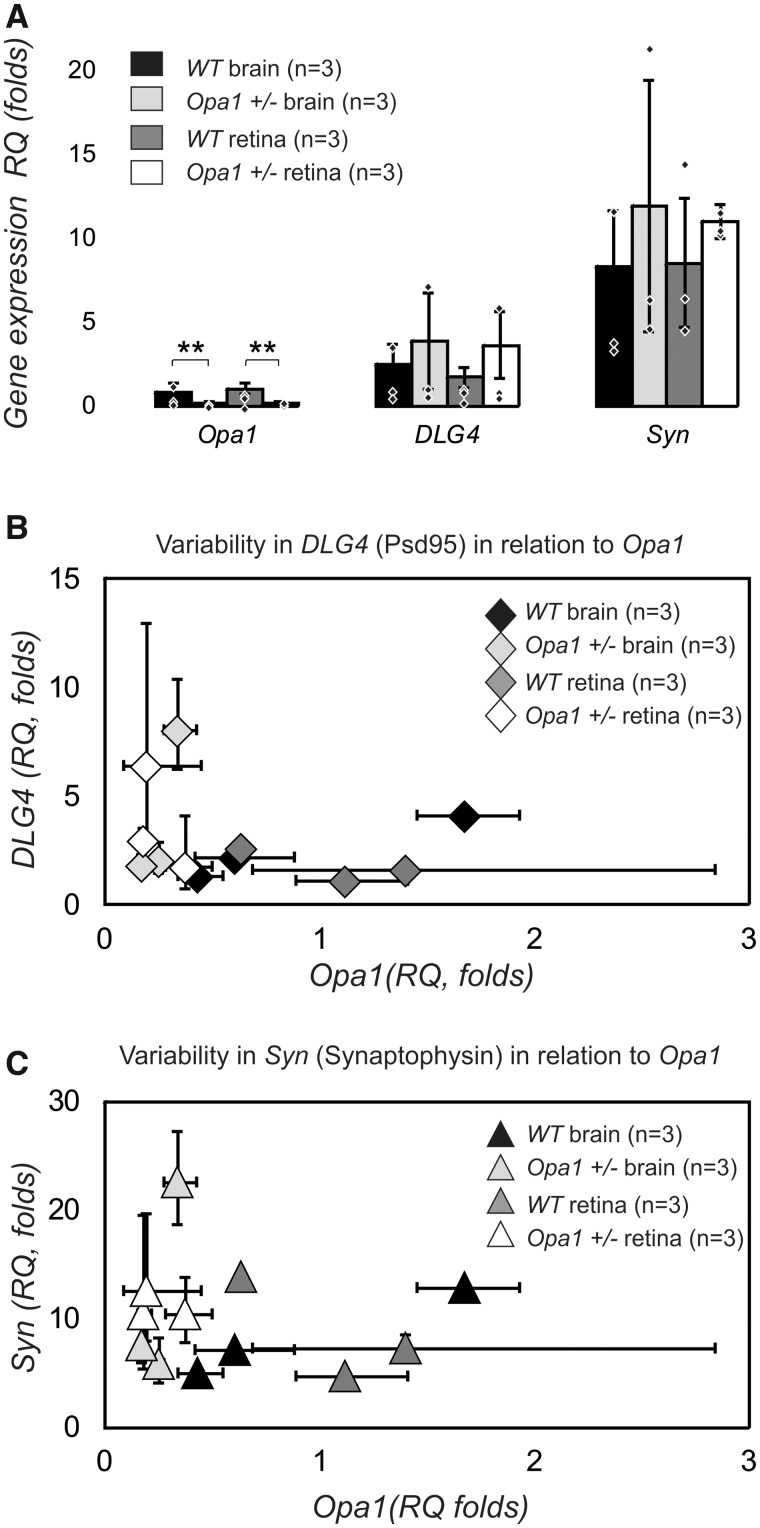
**Quantitative RT-PCR from 6 to 9 month old mice.** (**A**) Transcript levels of Opa1, DLG4 (encoding PSD95) and Syn (encoding Synaptophysin) in brain and retina of WT and mutant mice. Average for each subject is represented by individual point. There is a two folds reduction in *Opa1* gene transcript introduced by mutation. One-way ANOVA was performed on four groups of samples tested for each gene using ΔΔC values that were normally distributed. The average values are represented as RQ. (**B, C**) Analyses of variability of gene expression in individual samples for genes encoding for synaptic protein as a function of *Opa1* transcript. Analysis of relative gene expression data was performed using the 2-ΔΔCT method. The error bars represent max and min quantity of gene in a single biological sample. The low and upper error bars are not equal according to log transformation of ΔΔC values into RQ.

Quantitative RT-PCR was used to assess changes in transcript levels of *Opa1*, *Dlg4 (discs large homolog 4*; encoding for PSD95 protein) and *Syn* (encoding for synaptophysin protein) in the brain and retinas of WT and mutant mice at the age of 6–9 month prior to any detectable signs of neurodegeneration. Synaptophysin (*Syn* gene) and PSD95 (*Dl4* gene) are two synaptic proteins that are abundant in various types of tissue and dynamically change in ageing and disease. Their combination has been used in several recent studies both in animal and human tissue (Chen *et al.*, 2018; Liu *et al*., 2018) as a reliable synaptic marker (pre-synaptic and post-synaptic terminals are both labelled) to evaluate quantity and strength of synapses.We confirmed a two-fold reduction in *Opa1* transcript expression in the brain and retina in *Opa1* mutants (F-ratio 18.12; *P *<* *0.01; effect size *η*^2^ = 0.40 in retina and *η*^2^ = 0.46 in brain) Fig. 4A but no reduction in the expression of transcripts encoding for synaptic proteins PSD95 (F-ratio 2.07; *P* = 0.124) and synaptophysin (F-ratio 1.15; *P* = 0.345). Analyses of the variability of gene expression in individual samples (Fig. 4B and C) indicate that for both synaptic proteins, the levels of gene expression were relatively stable in WT animals, but less so in the mutants. Our data indicate that WT animals showed a broader range of *Opa1* gene expression with less variability compared to mutants.

#### Western blot

**Figure 5:**
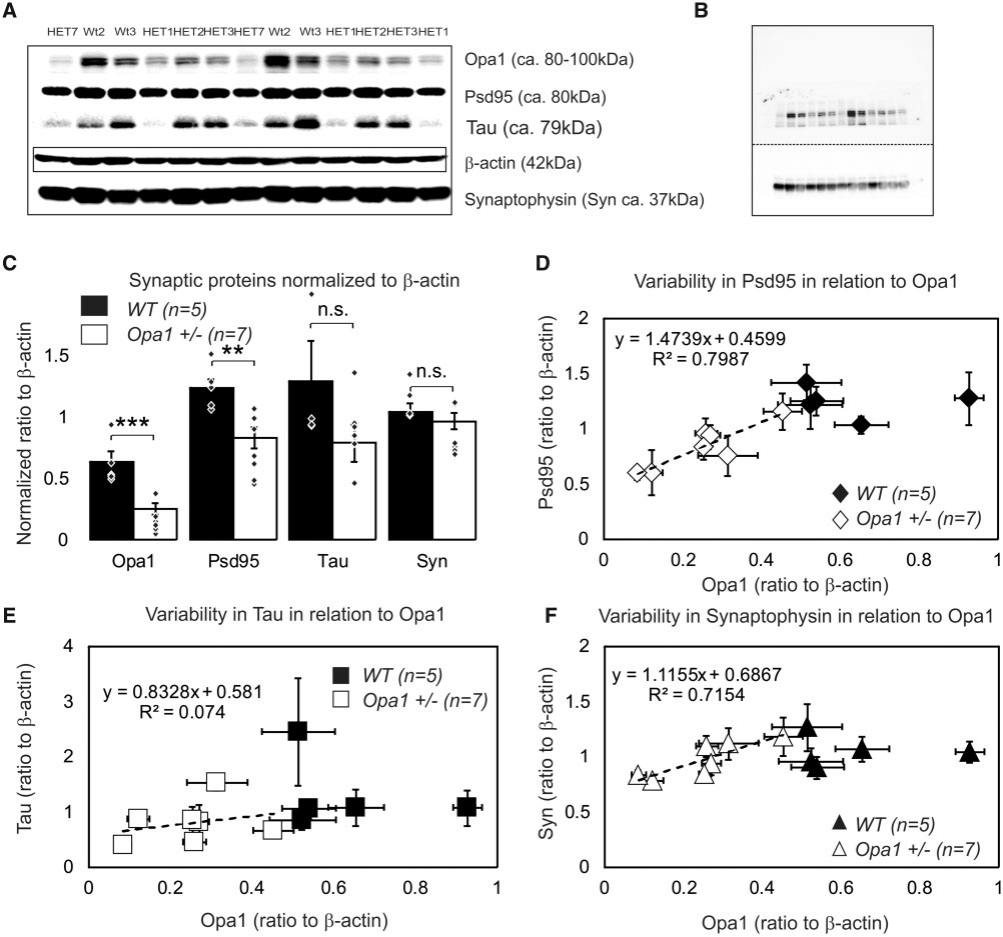
**Protein quantity assessment by western blot in 16 months old mice.** (**A**) An example of optimized western blot bands for gel (1 out of 6) containing 13 samples. The results shown in the panels A were obtained from 6 animals [2 WT and 4 *Opa1* (+/−)] and tested in triplicate. The experiment was repeated two more times (three biological samples per group) with smaller subset of proteins. All data were pooled together for **B**. Original (non-optimized) western blot membrane for gel 1 showing separately high molecular weight bands [OPA1 (top double band), PSD95, and Tau protein bands appearing simultaneously in close proximity] and low molecular weights bands (synaptophysin and β-actin). Row data are presented in Supplementary Fig. 5. (**C**) Average amount of proteins (normalized to β-actin; the *n* indicates number of independent biological samples). An average value for each independent biological sample is represented by individual point. All data are presented as mean and SE. *P*-values *<0.05, **<0.01, ***<0.01. (D–F) Variability in the level of relative expression of synaptic proteins in relation to levels of OPA1 protein in the sample. (**D**) PSD95. (**E**) Tau. (**F**) Synaptophysin. Data are represented as mean and SE of multiple repeats within the single biological sample.

In addition to previously mentioned synaptic proteins, we assessed microtubular stabilizing Tau protein levels. Tau presents in synapses and is abundant in axons. Correlating changes in Tau with changes in synaptic proteins allows distinguishing different scenarios of synaptic restructuring. For example, i) reduction in synaptophysin with no changes in other proteins would indicate reduced efficacy of pre-synaptic terminal; ii) reduction in synaptophysin with significant reduction in Tau would signal axonal degeneration and iii) PSD95 reduction in combination with modest decline in Tau would signal reduced size or postsynaptic terminals. We tested 12 biological samples with 4–6 individual repeats for 6 different proteins [mitochondrial OPA1 protein, post-synaptic protein PSD95, pre-synaptic protein synaptophysin, stabilize microtubules protein Tau, loading proteins β-actin (microfilaments) and voltage-dependent anion channel (a multi-functional mitochondrial protein)]. An example of a gel (1 out of 6) containing 13 samples is shown in Fig. 5A and B and Supplementary Fig. 5. The β-actin was found to be invariant to Opa1 levels (Supplementary Fig. 6) and was used as a normalization protein. As expected, OPA1 levels were reduced in *Opa1^+/−^* by ∼50%, *P *<* *0.001 (Fig. 5C). We detected a reduction in PSD95 (*P *=* *0.0015) and a statistically borderline reduction in Tau (*P *=* *0.058) proteins, but not Synaptophysin (*P *=* *0.20). Since OPA1 protein levels in individual samples showed large variability, we used it to correlate the levels of synaptic proteins with the levels of OPA1 (Fig. 5D–F). Regression analysis indicated that levels of PSD95 and synaptophysin (but not Tau) proteins were correlated with available OPA1 quantity.

In conclusion, we demonstrate that the mitochondrial fusion protein, OPA1, is essential for maintaining structural integrity of the neuronal dendritic and synaptic connections in the hippocampus. Consistent with dendritic changes in the hippocampus, learning and memory were also vulnerable to OPA1 deficiency, highlighting the importance of mitochondrial integrity in these processes.

## Discussion

Our behavioural data indicate previously unreported subtle learning and memory deficits in aged *Opa1^+/−^* mice. As *Opa1^+/−^* mice are visually compromised, we had a limited choice of suitable cognitive tests and were not able to determine the exact phenotype of cognitive impairment (Dere *et al.* 2005, 2007; Denninger, 2018). Ageing causes several well-documented behavioural changes including the decreased drive to explore. However, the validity of working memory as assessed by T-maze and object recognition tasks is well preserved with age (Ingram, 1988; Collier and Coleman, 1991; Fahlström *et al*., 2011). In our experiments, mutant animals showed not only hesitation to explore but also clear deficits in working memory tasks. Deteriorating vision is a hallmark of the *Opa1^+/−^* phenotype, in both male and female mice and we did not find any gender specific differences neither in magnitude nor in time course of vision loss. In contrast, working memory was affected differentially. A study by Brown and Wong (2007) suggested that mice with visual deficits were only disadvantaged when tested in highly visual tasks (like the Morris water maze), but had some additional advantages when tested in non-visual task (such as conditional order preference task). Moreover, another study by O’Leary *et al*. (2011), conducted in the dry maze, suggested that mice primarily use a non-visual search strategy, and learning and memory performance in a simple dry maze was not correlated with measures of visual ability. Indeed, similar memory tests (Wolf *et al*., 2016) are routinely used to demonstrate cognitive impairment in ageing models of various neurodegenerative diseases that also have documented visual deficits. We could not rule out that multiple brain regions were affected (Lisman *et al.*, 2017), because the behavioural strategy clearly contributed to the observed behavioural differences (Molas *et al.*, 2017). We focused our anatomical studies on the output hippocampal region CA1, since both T-maze and NOR tasks are hippocampus dependent (Tulving *et al.*, 1972).

In the hippocampus, synaptic proteins (especially PSD95) were downregulated, resulting in *Opa1*-depended remodelling at the postsynaptic site. Anatomical analysis of CA1 hippocampal region revealed shorter dendrites with reduced branching in female *Opa1^+/−^* mice. The spine density was reduced in both sexes, with proximal dendrites losing mostly stubby spines, and distant dendrites losing mostly mushroom spines. In addition, the aged female mice had more long thin spines compare to males, and these spines were all lost as a result of the mutation. Spine shape can reflect either different inputs or strength and stability of the synapse (Bourne and Harris, 2008). Synaptic changes were consistent with the observed cognitive deficits in *Opa1^+/−^* mice.

Mitochondrial dysfunction has emerged as an underlying feature of many neurodegenerative diseases. Mitochondrial fusion protein OPA1 is depleted in dendrites in hippocampal neurons in Alzheimer’s disease (Wang *et al.*, 2009). In the present study, we demonstrate that genetically encoded lower levels of OPA1 protein cause subtle anatomical changes in the CA1 hippocampal region (and possibly in other brain regions), at the level of spines and synapses. These changes are detrimental to learning and memory and resemble early structural changes found in Alzheimer’s animal models (Androuin *et al.*, 2018). In patients carrying *OPA1* mutations and having clinical signs of autosomal dominant optic atrophy, memory functions have not been systematically reported. However, cognitive deficits have been reported for *OPA1* mutations leading to Behr syndrome and Parkinson-like symptoms (Carelli *et al.*, 2015; Kleffner *et al.*, 2015; Rubegni *et al.* 2017).

Pyramidal neurons in the hippocampal CA1 region express OPA1 protein in both their soma and dendrites (Bette *et al.*, 2005). The observed reduction in the number of mature spines could have arisen either from reduced activity of pre-synaptic cells or/and from the failure of post-synaptic cells to maintain large spines and synapses. We cannot rule out the possibility that hippocampal deficits partially reflect sensory (visual) deprivation, as synaptic pruning in the retina of *Opa1^+/−^* mice was demonstrated by Williams *et al.* (2012) alongside with visual acuity deficits (Davies *et al.*, 2007; Barnard *et al.*, 2011). Homeostatic mechanisms routinely reshape dendrites and synapses based on ongoing activity. *In vivo* imaging of dendritic spines has shown a high degree of motility (Lendvai *et al.*, 2000). Changes in spines in adults are associated with long-term potentiation/depression (Engert and Bonhoeffer, 1999). Nevertheless, remodelling of spines and synapses may have undesired functional effects, including the disruption of networks that underpin memory (Mendez *et al.*, 2018). Von Bohlen Und Halbach *et al*. (2006) found that subtle changes in CA1 pyramidal cells, such as a decrease in spine numbers and length, correlate with behavioural deficits in learning tasks. Similar subtle changes are associated with neurological disorders such as schizophrenia and Down’s syndrome (reviewed in Fiala *et al.*, 2002), stress (Galea *et al.*, 1997), depression (reviewed in Qiao *et al.*, 2016) and anxiety (reviewed in Leuner and Shors, 2013).

Patient studies suggest that women are more at risk of developing dementia (Corfield, 2017) and their symptoms are more severe. We have previously reported a reduction in brain ATP levels in aged *Opa1^+/−^* mice (Smith *et al.*, 2016) that was more prominent for females. In the current study, female *Opa1^+/−^* mice were both slightly worse in behavioural tasks and displayed more changes in CA1 region. The shortening of CA1 pyramidal cell dendrites in female mice alone could account for poor T-test behavioural results (the test relies on the detection of spatial novelty). The distant apical dendrites of CA1 neurons receive direct input from the entorhinal cortex that may help to discriminate between different instances of memories for the same location (Remondes and Schuman, 2004; Zemla and Basu, 2017). In addition, a large number of long spines in aged female mice could indicate a general weakening of synaptic connections and hence weaker memory traces. A thin spine with a small head is likely to transduce a weaker signal than a stubby spine with a large head or a mushroom spine that can retain prolonged active status (Ebrahimi and Okabe, 2014). These differences could result from endocrine dysregulation. since hormones, especially oestrogen, affect dendritic arborization and spine density (Garrett and Wellman, 2009; Farrell *et al.*, 2015) and ovariectomized females display a drastic decline in spine density in the CA1 region (Gould *et al.*, 1990; reviewed in Frick *et al.*, 2015; Molly *et al.*, 2018). Oestrogen is known (Li *et al.*, 2004) to facilitate the spine-maturation process that supports hippocampal-dependent memory on a time scale of a few hours (Jacome *et al.*, 2016). Indeed, both males and females demonstrate improved behavioural score in memory tests when administrated female hormones (Frye *et al.*, 2005).

In the hippocampus, most excitatory synapses terminate on dendritic spines. Spines vary in size, with volumes that are proportional to the area of the post-synaptic density (PSD). PSD95 is a multi-domain postsynaptic scaffolding protein that clusters glutamate receptors and largely determines the size and strength of synapses. Although spines and their synapses can persist for months *in vivo*, synaptic proteins last only a few hours. Synapse stability requires a constant turnover of synaptic proteins with individual PSDs competing for a limited quantity of protein. Mature spines are more stable and can retain PSD95 for up to 5 h compared to median just under 2 h (Gray *et al.*, 2006). Our gene expression data suggest possible modulation of synaptic genes by *Opa1*. In control animals, the expression of genes encoding for synaptic proteins was stable, while expression of *Opa1* varied. The opposite was true in mutants; the *Opa1* was reduced but stable, while synaptic genes expression varied, adding to biological variability in the quantity of synaptic proteins. In addition, further age-related reduction in OPA1 protein caused a persistent deficit in the availability of PSD95 and thus increased competition between individual synapses. Cognitive impairment in mitochondrial diseases (reviewed by Finsterer, 2012; Picarda and McEwen, 2014; Khacho *et al.*, 2017) is increasingly recognized and diagnosed as mitochondrial cognitive dysfunction (MCD). Variability in the onset and severity of inherited diseases (including dementias) often means that the same family mutation can generate different clinical histories and prognoses.

From the clinical point of view, testing memory function in patients harbouring *OPA1* mutations could uncover subtle deficits that would facilitate genetic diagnosis and establish new disease phenotypes. Further work is required to determine the influence of, natural variation in OPA1 levels on cognitive function in healthy ageing.

## Supplementary material

Supplementary material is available at *Brain Communications* online.

## Supplementary Material

fcaa101_Supplementary_Data

## Abbreviations

Actin (actin) =: β-actin isoform of multi-functional protein that form microfilameints
ANOVA =: analysis of variance, a collection of statistical models and their associated estimation procedures to analyse the differences among group means in a sample
ARVO =: The Association for Research in Vision and Ophthalmology
ATP =: adenosine triphosphate, the energy-carrying molecule that can release energy very quickly
CA1 =: The region in the hippocampal circuit, from which a major output pathway goes to the entorhinal cortex
Ct (in qPCR) =: cycle threshold
DI =: discrimination index
DiOlistics =: cell labelling technique
DNA =: deoxyribonucleic acid
EC =: European Commission
EMPReSS =: European Mouse Phenotyping Resource for Standardized Screens
HET =: heterozygote, Opa1 +/− genotype, has only one copy of Opa1 gene, in contrast to wild type that has two copies of Opa1 gene
NOR =: novel objects recognition, behavioural test of memory
OKN =: optokinetic response, a reflex to track a moving object with eyes or body
OPA1 =: optic atrophy 1 (OPA1 human gene, Opa1 mouse gene, OPA1, associated protein)
PSD95 =: post-synaptic density protein 95, a postsynaptic scaffolding protein in excitatory neurons
qPCR =: quantitative polymerase chain reaction
RI =: recognition index
RIPA buffer =: radioimmunoprecipitation assay buffer
RNA =: ribonucleic acid
RT-PCR =: reverse transcription polymerase chain reaction, a laboratory technique combining reverse transcription of RNA into DNA and amplification of specific DNA targets
RQ (in qPCR) =: relative quantification = 2-ΔΔCt
SDS =: sodium dodecyl sulphate
SPSS =: Statistical Package for the Social Sciences, IBM SPSS^®^ is a statistical software
Tau =: tau protein, maintains stability of microtubules in axons, abundant in neurons
TB, TN, TF =: time spent at the base of the maze/testing arena, and exploring novel and familiar environment/object, respectively
T-maze =: T-shaped maze, used for behavioural test of memory
VDAC =: voltage-dependent anion channel, or mitochondrial porin, membrane protein located on the outer mitochondrial membrane
WT =: wild type, Opa1 +/+ genotype, has two copies of Opa1 gene
