# Supplementary material for: OPA1 deficiency accelerates hippocampal synaptic remodelling and age-related deficits in learning and memory
Source: Brain Commun. 2020 Jul 15;2(2):fcaa101. doi: 10.1093/braincomms/fcaa101 (PMC7566495; doi:10.1093/braincomms/fcaa101)
Supplement: fcaa101_Supplementary_Data [file fcaa101_supplementary_data.pdf]

Supplementary Figure 1. Meta-analyses of mouse visual acuity (OKN behavioural data) time spent tracking 0.25 cycles per degree stimulus

		N
Sex	female	52
	male	71
Age (months)	6	38
	12	35
	14	38
	20	12
Genotype	*Opa1+/-* (HET)	61
	WT	62

Tests of Between-Subjects Effects

Dependent Variable: Time Tracking

Source	Type III Sum of Squares	df	Mean Square	F	Sig.
Corrected Model	6124.430[a]	15	408.295	2.667	0.002
Intercept	57170.489	1	57170.489	373.415	0.000
Sex	137.767	1	137.767	0.900	0.345
Age	1130.472	3	376.824	2.461	0.067
Genotype	1899.794	1	1899.794	12.409	0.001
Sex * Age	148.969	3	49.656	0.324	0.808
Sex * Genotype	179.903	1	179.903	1.175	0.281
Age * Genotype	2118.082	3	706.027	4.611	0.004
Sex * Age* Genotype	74.889	3	24.963	0.163	0.921
Error	16381.887	107	153.102		
Total	100076.381	123			
Corrected Total	22506.317	122			

a. R Squared = 0.272 (Adjusted R Squared = 0.170)

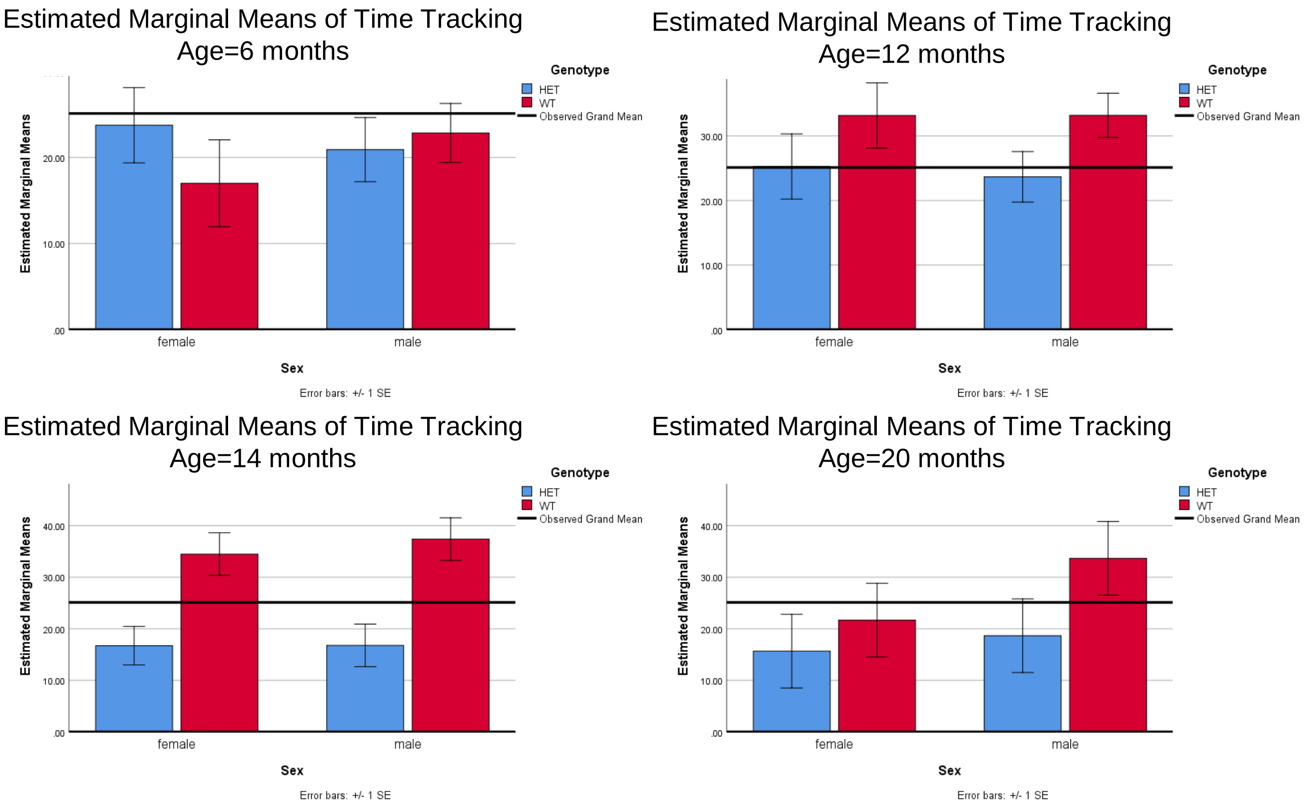

Supplementary Figure 1. Summary of meta-analyses of mouse visual acuity tests (OKN behavioural data). The data were combined from the experiments conducted in this study and the earlier experiments performed in the same behavioural laboratory with the same methodology and previously reported by Davis et al., 2007. The first table specifies the number of animals used for this analysis grouped by gender, age, and genotype. The second table shows results of multivariant ANOVA on combined sample of 123 subjects where between-subjects' factors (gender, age, and genotype) were analysed. The genotype emerged as the most significant factor, followed by age/genotype interaction. From the tests conducted, gender does not seem to influence severity or time course of vision impairment. The bar chart below shows estimated means of time spent tracking the stimulus grouped by gender, age, and genotype. The black line shows global mean of this variable for all available subjects. Please, note, that absolute values change with age and are not directly comparable between the age groups. All data analysis was performed using SPSS statistic package (IBM, US).

Supplementary Figure 2.
Additional parameters measured during behavioural memory tests

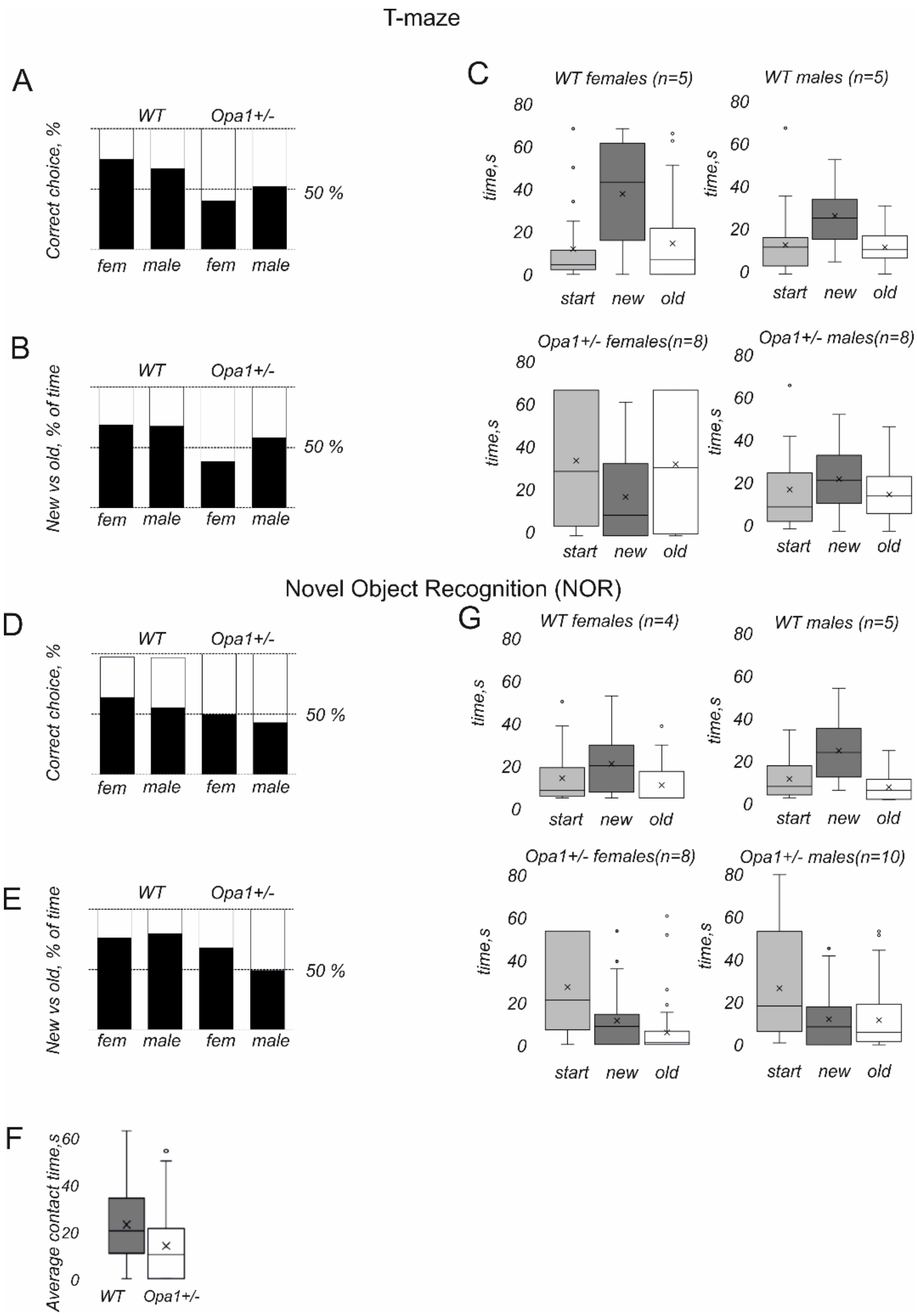

Supplementary Figure 2. Summary of behavioural data characterising learning and memory (to complement Fig.1) A-C. T-maze test. D-G. Novel Objects Recognition (NOR) test. A. Percentage of correct choices in T-maze task (when the arm choice was considered to be correct), grouped by gender and genotype. B. Percentage of total exploration time spent exploring novel T-maze arm, grouped by gender and genotype. C. Full descriptive statistics (represented by whisker plots, with median marked as a line across a bar, mean shown as a cross, and outlays shown as circles) detailing variability in exploratory behaviour. For each gender and genotype 3 time variables are shown: 1) time spent at the base of the maze (start), 2) time spent exploring novel T-maze arm (new), and 3) time spent exploring familiar T-maze arm (old). D. Percentage of correct choice in NOR task (the novel object was considered to be correct), grouped by gender and genotype. E. Percentage of time spent exploring novel object in NOR task vs total time spent exploring objects, grouped by gender and genotype. F. Average object contact time grouped by genotype. G. Full descriptive statistics (represented by whisker plots, with median marked as a line across a bar, mean shown as a cross, and outlays shown as circles) detailing variability in NOR exploratory behaviour. For each gender and genotype 3 time variables are shown: 1) time spent hesitating before first approaching an object (start), 2) time spent exploring novel object (new), and 3) time spent exploring familiar object (old). Each subject was tested multiple times, and all data were used. The data were treated as nested data (see Methods) and effective sample size was calculated based on variability between different trials and variability between subjects.

Supplementary Figure3.
Examples of spine tracing using **Imaris** software

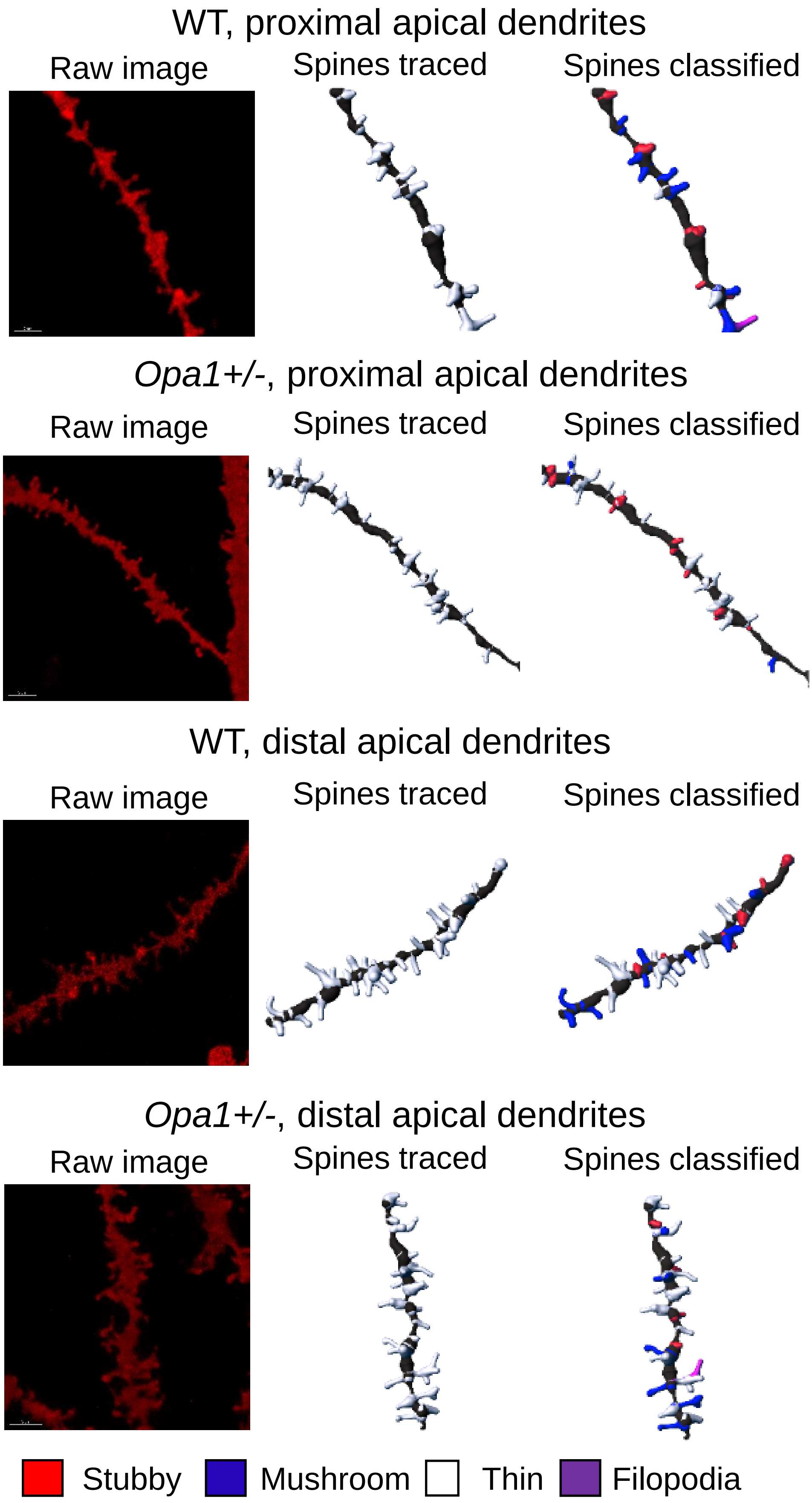

Supplementary Figure 3. Examples of spine tracing using Imaris (Bitplane, Switzerland) software. Representative examples are shown for distal and proximal dendrites of CA1 pyramidal cells for each of genotypes. Left panels show confocal images. Central panels show the same dendrites with identified spines. Right panels show the same dendrites with color-coded classified spines (red-stubby, blue-mushroom, white–thin, and purple –filopodia).

Supplementary Figure 4. Automatic vs manual spine classification

An example dendrite from *WT* male (Fig3)

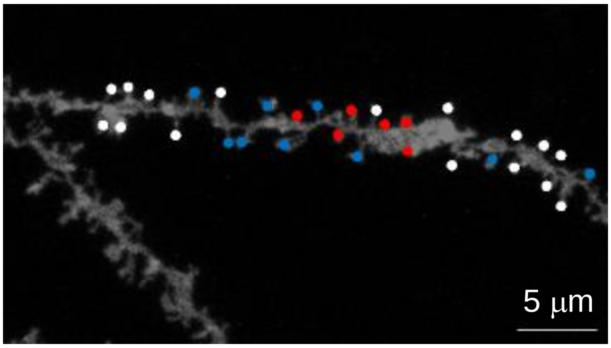

Manual:

Number of spines	31
Stubby	6 (red)
Mushroom	9 (blue)
Thin	16 (white)

Automated:

Number of spines	30
Stubby	4 (red)
Mushroom	10 (blue)
Thin	16 (white)
Correctly Classified	93.5% (29/31)

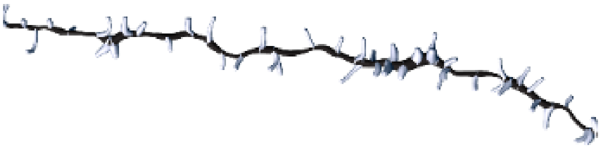

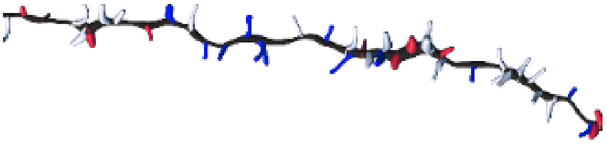

An example dendrite from *Opa1* +/- male (Fig3)

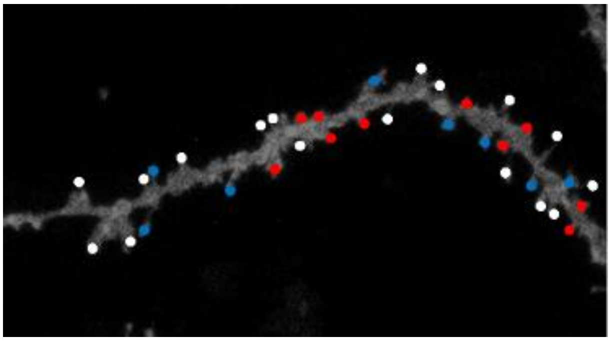

Manual: 35

Number of spines	35
Stubby	10 (red)
Mushroom	8 (blue)
Thin	17(white)

Automated: 35

Number of spines	35
Stubby	8 (red)
Mushroom	9 (blue)
Thin	18 (white)
Correctly Classified	94.2% (33/35)

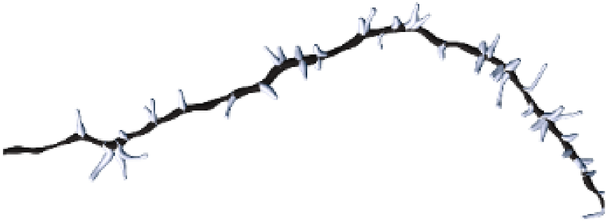

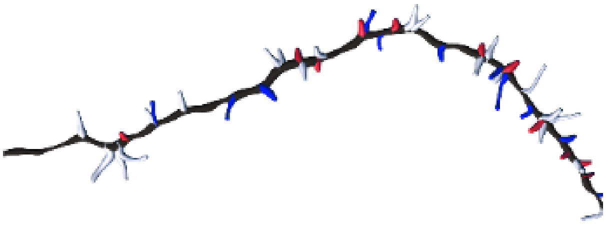

Supplementary Figure 4. Examples of automatic (Imaris) vs manual spine classification for two representative examples shown in Fig. 3. Upper panel shows analysis for a dendrite from WT male subject, and the lower panel shows identical analyses for HET male. Both methods were in good correspondence with each other and there were no bias introduced by genotype.

Supplementary Figure 5.
Western Blot gel with Actin, Synaptophysin, Tau, OPA1, and PSD95

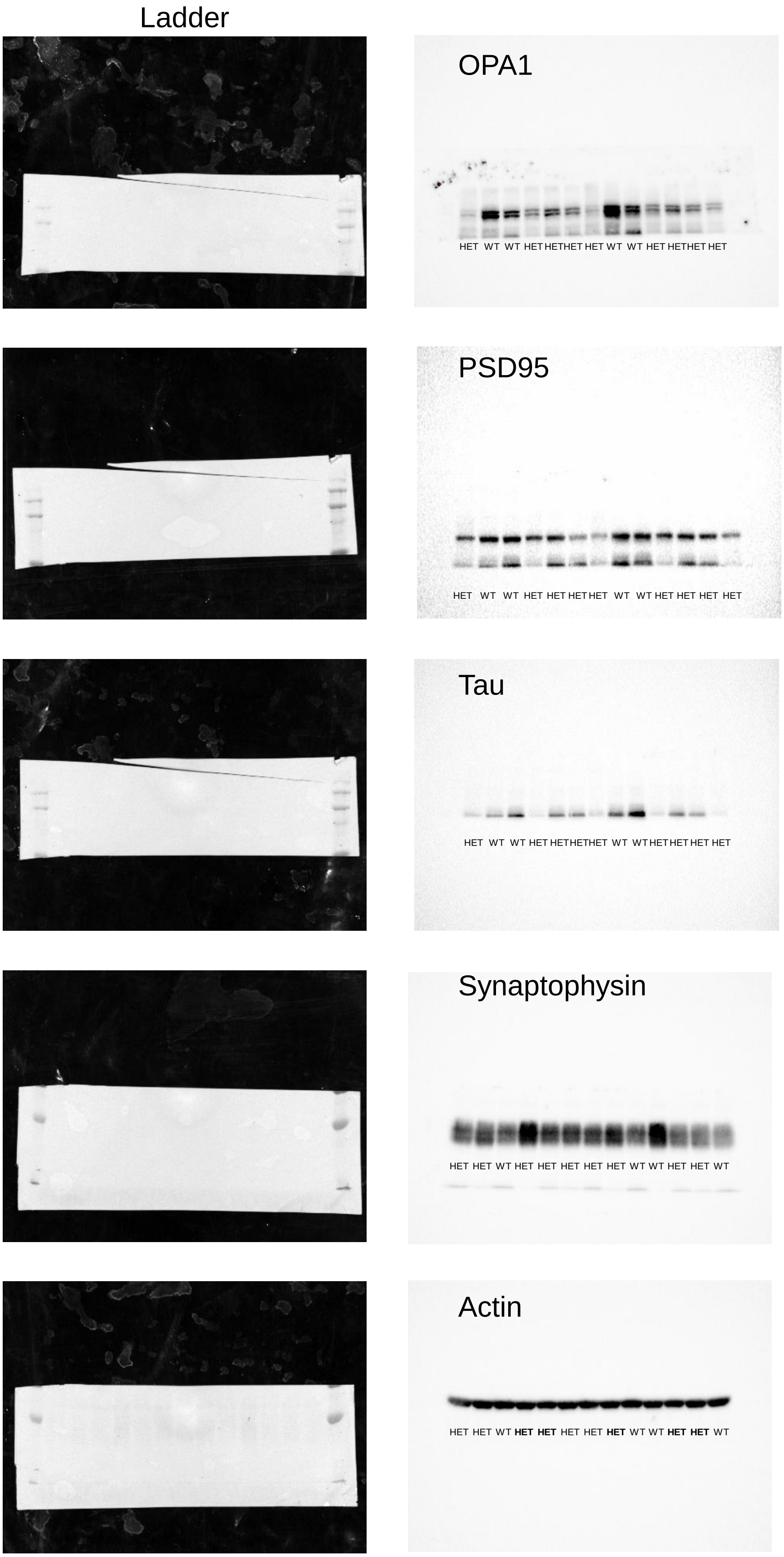

Supplementary Figure 5. Western blot gel (from Fig.5) with Actin, Synaptophysin, Tau, OPA1, and PSD95. Western blot membrane was cut and is shown separately for high molecular weight bands (OPA1 (top double band), PSD95, and Tau protein bands appearing simultaneously in close proximity) and low molecular weight bands bands (Synaptophysin and β-actin).

Supplementary Figure 6.
Loading proteins Actin and VDAC as a function of OPA1 protein level

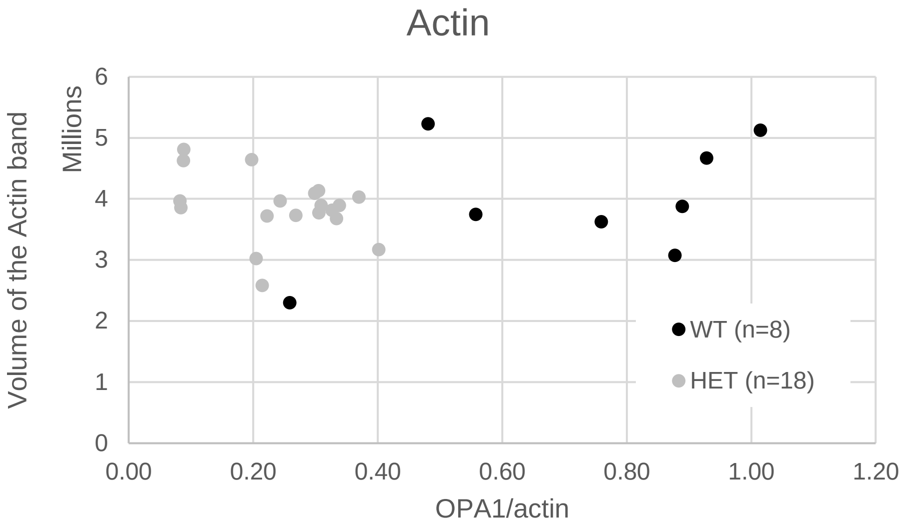

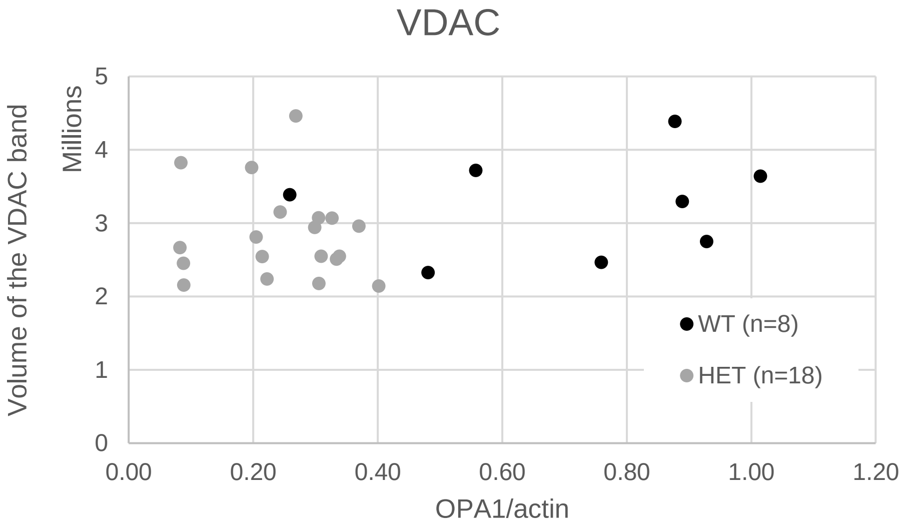

Supplementary Figure 6. Western blot loading proteins β-actin and VDAC. Upper and low plots show total volume of bands representing β-actin (upper plot) and VDAC (lower plot) as a function of normalised to β-actin OPA1 protein for 26 samples tested (both genotypes). Both loading proteins β-actin and VDAC did not show any OPA1-dependence. The β-actin was chosen for analyses because its band has a lower molecular weight and it is clearly separated from the bands representing target synaptic proteins.

List of abbreviations used in Supplemental materials:

Actin (actin)	β-**actin** isoform of multi-functional protein that form microfilaments.
ANOVA	**An**alysis **o**f **va**riance, a collection of statistical models and their associated estimation procedures to analyse the differences among group means in a sample.
CA1	The region in the hippocampal circuit, from which a major output pathway goes to the entorhinal cortex.
HET	**Het**erozygote, *Opa1 +/-* genotype, has only one copy of *Opa1* gene, in contrast to wild type that has two copies of *Opa1* gene.
NOR	**N**ovel **O**bjects **R**ecognition, behavioural test of memory.
OKN	**O**pto**kin**etic response, a reflex to track a moving object with eyes or body
PSD95	**P**ost**s**ynaptic **d**ensity protein 95, a postsynaptic scaffolding protein in excitatory neurons.
SPSS	**S**tatistical **P**ackage for the **S**ocial **S**ciences, IBM SPSS® is a statistical software
Tau	**Tau** protein, maintains stability of microtubules in axons, abundant in neurons
T-maze	**T** shaped **maze**, used for behavioural test of memory.
VDAC	**V**oltage-**d**ependent **a**nion **c**hannel, or mitochondrial porin, membrane protein located on the outer mitochondrial membrane.
WT	**W**ild **T**ype, *Opa1 +/+* genotype, has two copies of *Opa1* gene.
